# Antral contractility following Asian-style meal in healthy volunteers: effect of genders, menstruation, smoking status and age

**DOI:** 10.22038/AOJNMB.2021.60453.1423

**Published:** 2022

**Authors:** Janyarut Srijumruen, Sira Vachatimanont, Usanee Techavijit, Tawatchai Chaiwatanarat

**Affiliations:** 1 Buddhachinaraj Phitsanulok Hospital, Phitsanulok, Thailand; 2 Nuclear Medicine Division, Department of Radiology, Chulalongkorn University and King Chulalongkorn Memorial Hospital, Thailand; 3 Nuclear Medicine Division, Department of Radiology, Chulalongkorn University, Rama IV Rd, Pathumwan, Bangkok, Thailand

**Keywords:** Gastrointestinal Motility, Healthy volunteers, Radionuclide imaging, Antral contractility

## Abstract

**Objective(s):**

The normal range of the frequency of antral contractions, similar to other gastric motility parameters, are different depending on the population and the test meal. We, therefore, conducted the analysis to establish the normal ranges for the frequency of antral contractions derived from dynamic antral scintigraphy (DAS) following an Asian-styled solid meal in Thai healthy volunteers.

**Methods:**

We retrospectively analyzed the data from the study on normal gastric emptying values. The dynamic scintigraphic images had been obtained in a 45 degree left anterior oblique view during the first 32 minutes following the ingestion of a 267-kcal steamed rice, a technetium-99m-labeled microwaved egg and 100 mL of water. A polynomial normalization model was used to analyze and to calculate the frequency of antral contractions. The data analysis was repeated by the same operator 1 month apart to assess reproducibility.

**Results:**

Data of 18 volunteers (10 males and 8 females) were eligible for analysis. The mean±SD of the frequencies of the antral contractions were 3.06±1.08 cycles/min on the first and 3.04±1.10 cycles/min on the second analysis. Comparison of the two analyses revealed a moderate agreement (ICC=0.632, 95% CI 0.329-0.818) without significant difference (p-value=0.942). No significant effect of gender, menstruation status, smoking or age was demonstrated in this study.

**Conclusion:**

DAS is a non-invasive technique that can measure the frequency of antral contractions. The technique is reproducible and consistent. Future study may be required to assess the effect of gender, menstruation status, smoking and age.

## Introduction

 Gastric and intestinal motility disorders affect quality of life in a large number of the population worldwide. They are responsible for a considerate amount of medical expenses. Apart from global delay in gastric emptying, antral hypomotility is another abnormal motor function found in several gastric motility disorders (1). It was reported not only in gastroparesis but also in functional dyspepsia and idiopathic nausea and vomiting (2–4). 

 Because the symptoms of these disorders are usually discordant with the severity of the motility dysfunction, the measurements of gastric motility are useful in guiding the treatment strategies (1).

 The antral motility is traditionally measured by intraluminal manometry, which is an invasive procedure. Thus, several non-invasive testing, one of which is the dynamic antral scintigraphy (DAS), has been developed to provide the alternative non-invasive method of

 antral contractility measurement (4).

 Because the global gastric emptying differs by gender, race, the texture and energy of the test meals, it is possible that the antral motility is also affected by similar factors. There have been evidences from previous studies that antral contraction frequency is probably higher in females than males. Higher frequency has also been reported following solid than liquid meal (3, 5).

 Because there has been no study that provided the normal values of antral contraction frequency in Asian population following Asian-style meals. We conducted the current study to measure antral contractility from DAS in Thai healthy volunteers following an Asian rice-based solid meal.

## Methods

***Subjects***

 The current study was a retrospective analysis of the prospectively collected data. The data retrieval and data analysis were approved by Institutional Review Board of Faculty of Medicine, Chulalongkorn University (IRB No. 513/58). The subjects comprised a subset of 26 Thai healthy volunteers from King Chulalongkorn Memorial Hospital. All volunteers aged above 18 years and were recruited for measurement of normal gastric emptying values in the study by Vasavid et al. and had been consented of data collection for further analysis (6). Any volunteers with gastrointestinal symptoms, gastroenteritis within 3 months or history of abdominal surgery were excluded.

 The subjects were subgrouped according to smoking status. Any volunteers who smoked at least a cigarette a day for more than a year were defined as smokers. Any subjects who smoked at least 1 cigarette a day for more than a year but had quitted for at least one year before the study were classified as ex-smokers. Any smokers must stop smoking at least 24 hours before the study.

 The female subjects were further subgrouped based on menstruation status. A subject whose study date was in the last 14 days of the menstrual cycle was considered to be in the luteal phase. A postmenopausal woman or a subject whose study date was before the last 14 days of menstrual cycle was considered to be in the non-luteal phase. Subjects with irregular cycle or unreliable menstruation history were grouped together as unclassified.

***Test meal***

 The test meal consisted of 100-g steamed rice, a radiolabeled microwaved egg and 100 mL of water. The egg was prepared from 65-70 g whole egg that was mixed with 5 mL vegetable oil, 10 mL of water and 37 MBq of Tc-99m phytate or Tc-99m sulfur colloid. The egg mixture was later scrambled and boiled in a microwave. The total calorie of the meal was 267 kcal (57% carbohydrate, 23% fat and 19% protein). The subjects were allowed to modify the taste of the meal by sauce containing mainly soy and salt. The sauce contained neither calorie nor carbohydrate content.

***Image acquisition***

 After at least 6 hours of fasting, the subject ingested the test meal within 10 minutes. The images were acquired using a gamma camera (Symbia T6, Siemens, Erlangen, Germany). The camera was set to record activity with a 20% window around the 140 keV photopeak of Tc-99m. The 128×128 matrix images were acquired using a single head of gamma camera equipped with general purpose collimator. One hundred and fifty 2-sec/frame dynamic images were acquired in anterior upright position 32 minutes after the ingestion of the test meal. Total subjects were instructed not to talk or move during the image acquisition.

***Image processing***

**Figure 1:**
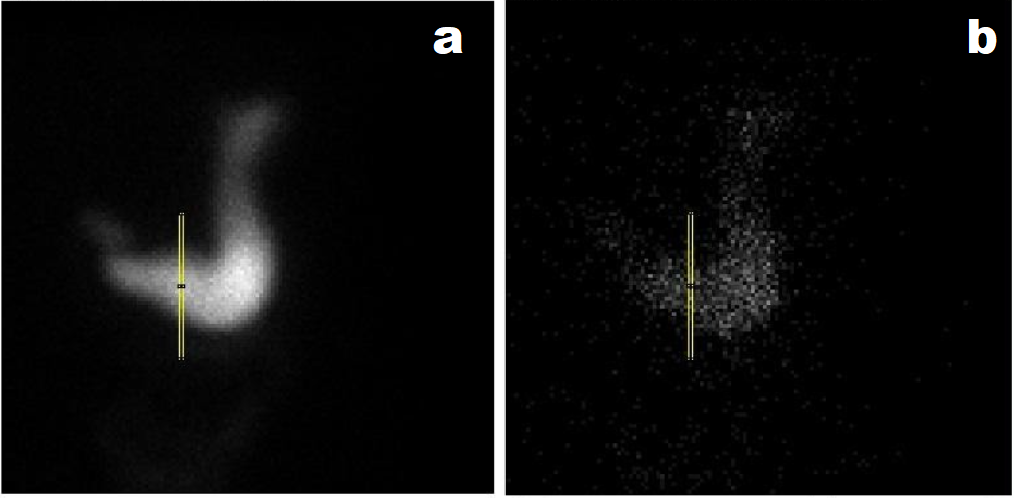
A rectangular ROI drawn at the most horizontal part of the gastric antrum displayed on composite (**a**) and dynamic (**b**) images

 One hundred and fifty 2-second images acquired at 32 min were summed up to form a composite image. Over the composite image, a one-pixel-width rectangular ROI was drawn at the most horizontal part of the gastric antrum at a mid-distance between the pylorus and incisura angularis (Figure 1) (3, 5, 7). An activity-time curve (ATC) in the ROI, representing the antral activity, was then created.

**Figure 2:**
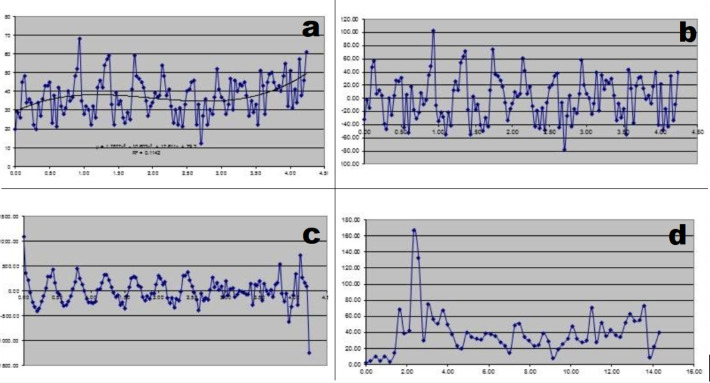
Image processing. (**a**) raw data with 3rd degree polynomial curve fitted. (**b**) ATC after normalization (**c**). ATC after autocorrelation and (**d**) result of fast Fourier analysis

 To express activity as a percentage of the mean counts, polynomial normalization of the ATC was performed. The 3rd order polynomial, equation 1, was fit to the raw data curve (Figure 2a-2b). Then the normalized activity (Anormalized) of the raw activity (Araw) at each time point (t) was calculated from equation 2:

*Y = ax3+bx2+cx+                                                                                      *(1)

*Anormalized= (Araw – (a.t3 + b.t2 + c.t + d)) / d x 100*           (2) 

 The autocorrelation function was then applied to the normalized curve to decrease noise signals (Figure 2c) (4, 5). The resulting curve was processed by spectral analysis using the fast Fourier transform. The frequency of the antral contractions, referred in the current study as antral frequency, was defined as the frequency with the highest Fourier power in the

 frequency spectrum (Figure 2d).

 The commercial software Microsoft Excel version 2010 was used for the fast Fourier analysis of the data. Intra-observer variability was assessed by repeating data analysis by the same operator one month apart.

***Statistical analysis***

 Frequencies of antral contractions were reported as mean and standard deviation. Intra-observer reliability was measured by intraclass correlation coefficient (ICC). Comparison of continuous variables was performed by t-test. P-value<0.05 was considered statistically significant. All data were analyzed using the R statistical packages (RStudio).

## Results

***Baseline characteristics***

 A total of 18 subjects were included into the analysis. The demographic data and characteristics of the subjects are displayed in Table 1.

**Table 1:** Baseline characteristics of eligible subjects

Demographic data	Total	Male	Female
1. Gender; n (%)	18 (100)	10 (56)	8 (44)
2. Age; years (Mean±SD)	40.67±13.32	39.30 ± 11.79	42.38±15.69
3. Weight; kg (Mean±SD)	60.78±10.59	64.45±9.75	56.20±10.33
4. Height; cm (Mean±SD)	162.56±7.06	167.00±5.10	157.00±4.90
5. BMI; kg/m2 (Mean±SD)	22.99±3.85	23.06±3.07	22.91±4.89
6. Smoking			
Non-smoker; n (%)	13 (72%)	5 (50%)	8 (100%)
Smoker; n (%)	4 (22%)	4 (40%)	-
Ex-smoker; n (%)	1 (6%)	1 (10%)	-
7. Menstrual status			
Menopause; n (%)	-	-	1 (13.5%)
Follicular phase; n (%)	-	-	3 (38.5%)
Luteal phase; n (%)	-	-	2 (25%)
Unclassified; n (%)	-	-	2 (25%)

***Intra-observer variablity***

**Figure 3:**
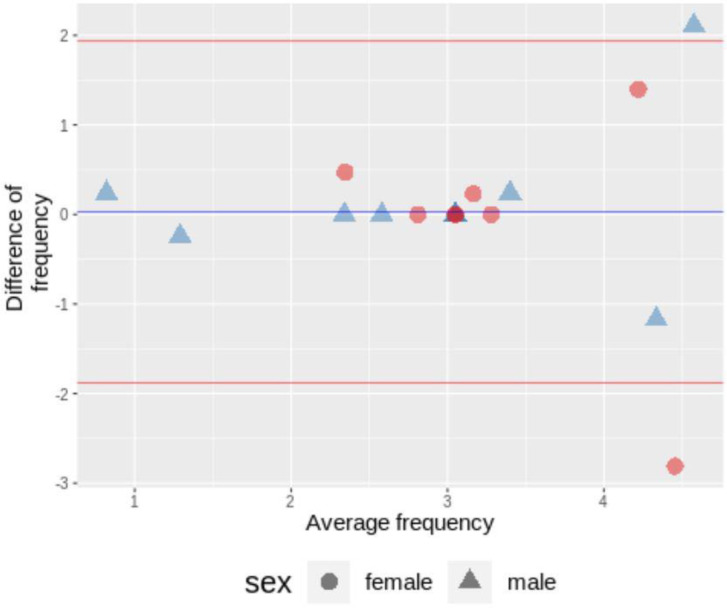
A Bland-Altmann plot showing average and difference between antral frequency from the first and the second analyses

 The mean±SD of antral frequency of the 2 analyses were 3.06±1.08 and 3.04±1.10 cycles/min, respectively. There was a moderate agreement (ICC=0.632, 95% CI 0.329-0.818) with no statistical significant (p-value = 0.944) difference between the two analyses (Figure 3).

***Effect of gender and menstruation on antral contractility***

**Figure 4:**
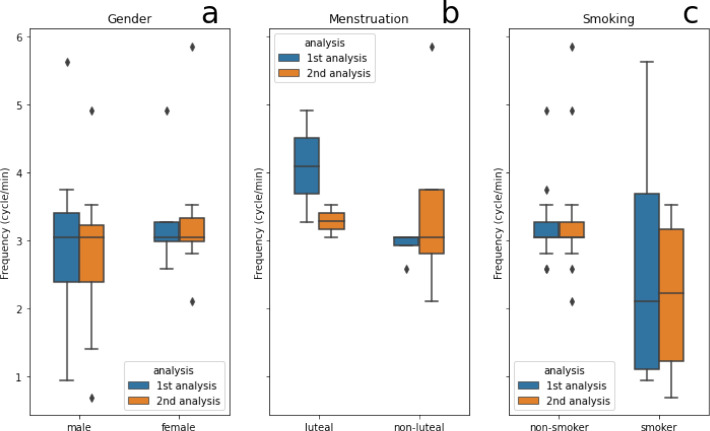
Box plots showing antral frequencies, compared between genders (**a**), menstruation (**b**) and smoking status (**c**)

 In the first analysis, the mean±SD were 2.91±1.33 cycles/min in male and 3.25±0.71 cycles/min in female. In the second analysis, the mean±SD were 2.79±1.16 cycles/min in male and 3.34±1.10 cycles/min in female. There was no significant difference between male and female on either the first (p-value=0.519) or the second (p-value=0.319) analyses. (Figure 4a) Between the two analyses, there was a good agreement of the male (ICC=0.805, CI 0.508-0.933) and a poor agreement of the female (ICC=0.221, 95% CI -0.381-0.708) data.

 Regarding menstruation status, in the first analysis, the mean±SD of antral frequency were 4.10±1.16 cycles/min in luteal female and 2.93±0.23 cycles/min in non-luteal female. In the second analysis, however, the mean±SD were 3.28±0.33 cycles/min in luteal female and 3.52±1.62 cycles/min in non-luteal female. We found no significant difference between luteal and non-luteal females on the first (p-value=0.093) or the second (p-value= 0.859) analyses. The agreement between the two analyses was poor in both luteal (ICC=0.377, 95% CI -0.787-0.995) and non-luteal female (ICC=0.213, 95% CI -0.621-0.867) (Figure 4b).

***Effect of smoking on antral contractility***

 The antral frequencies of smokers (1st analysis: 2.70±2.17 cycles/min, 2nd analysis: 2.17±1.33 cycles/min) were slightly lower than non-smoker (1st analysis: 3.23±0.61 cycles/min, 2nd analysis: 3.35±0.99). There was, however, no statistical significance difference on either analysis (1st analysis: p-value= 0.417, 2nd analysis: p-value= 0.071). The agreement between the two analyses was moderate in non-smoker (ICC=0.716, 95% CI 0.075-0.948) and good in smoker (ICC=0.824, 95% CI 0.223-0.979) (Figure 4c).

***Effect of age on antral contractility***

**Figure 5:**
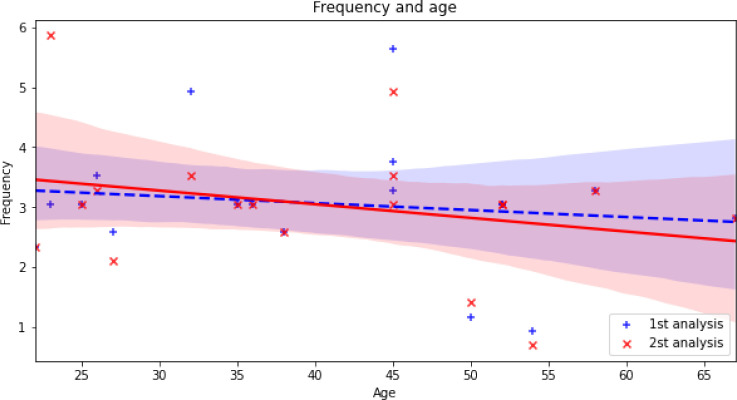
Simple linear regression between antral frequencies and ages

Figure 5 shows the results of simple linear regression between antral frequencies and ages. We suspected a trend of lower antral freque-ncies in older subjects.

(1st analysis: R2=-0.143, 2nd analysis: R2=-0.267) However, the trend failed to achieve statistical significance (1st analysis p-value = 0.573, 2nd analysis: p-value=0.284).

## Discussion

 In this study, we demonstrated antral frequency following Asian-style solid meal in Thai healthy volunteers. Establishing expected ranges of antral frequency in normal subjects for specific meals and population is crucial because the normal values of gastric motility parameters are influenced by characteristics of test meals and subjects.(6,8) We found that the mean±SD of antral frequency of the 2 analyses were 3.06±1.08 and 3.04±1.10 cycles/min. Our frequencies were higher than 2.78±0.18 cycles/min, which was the antral frequency following liquid meals from the study of Misiara et al. in 2007(3). 

 Regarding the effect of gender, we found no significant difference in antral frequency between male and female. Our result was concordant with the result of Bennick et al. (9) who also found no significant difference of antral contractility between male and female. Conversely, Knight et al. (5) demonstrated higher antral frequency in female than male. The effect of gender on antral contractility is theoretically possible and may be explained by the difference in sex hormone responsiveness among regions of gastric wall.

 Smoking may be a potential factor leading to the decrease in the contractility of the gastric antrum despite the fact that we found no significant different antral frequencies between smokers and non-smokers. Because the number of smokers among our subjects was low (n=4), it is possible that in larger sample size, a statistical significant difference may be able to achieve. It had been demonstrated that smoking was a factor leading impaired gastric emptying (10), and smoking cessation led to the acceleration of gastric emptying (11). The same mechanism may be responsible for both the decrease in antral contractility and the impairment of gastric emptying. 

 There have been a few evidences on the trend towards lower antral frequency in older subjects, even though not conclusive. In 1983, Moore et al. (12) found that there was a delay in liquid, but not solid, emptying in older subjects. In 1984, however, Horowitz et al. (13) found the slowing of both liquid and solid emptying in older subjects. Those were different from recent study in 2004, in which Madsen et al. (14) found that the gastric motility was not affected by age.

Clinical applications of gastric motility measurement can be very diverse because abnormal gastric motility is found in a large variety of diseases. In 2000, Linke et al. (15) demonstrated delayed gastric emptying in patients with scleroderma and diabetes mellitus. In 2006, Troncon et al. (8) found a delay in gastric emptying among patients with functional dyspepsia. 

 One potential application of AS is to differentiate subgroups of functional dyspepsia. Recently in 2012, Ahmed et al. (16) evaluated antral contractility using endoscopic ultrasonography and found significant different antral contractions between two subgroups of functional dyspepsia: postprandial distress syndrome and epigastric pain syndrome. Because the two subgroups requires different treatments, (17) DAS can be a non-invasive tool offering patients the correct diagnosis and management.

 There are several limitations to our study. First, the number of subjects was low. This can lead to failure in detection of subtle differences in antral frequencies between subgroups. Secondly, all smokers were male and there has been no evidence to infer that smoking affects antral contraction in male and females in the same manner. Thirdly, a reliable menstruation history was not obtained in a considerable fraction of female subjects (n=2, 25% of female subjects), causing a gap of missing data for analyzing the effect of menstruation. Fourthly, the subjects were allowed to modify the taste of test meal. We assumed the effect of the seasoning was unlikely because the volume was small, the pH was neutral and the major factors influencing gastric emptying were the physical distension and the pH (18). Despite the aforementioned reasons, we could not exclude the possibility of altered antral contractions resulting from taste modification. Finally, we examined only the frequency of antral contractions, leaving others aspects, such as the amplitude, for future investigation.

## Conclusion

 In conclusion, the current study is the first to describe the frequency of antral contractions following a solid Asian-style meal in healthy adults using DAS. We proposed that measuring the frequency using DAS is reproducible. No significant effect of gender, menstruation status, smoking or age was demonstrated in the current study but studies with a larger number of subjects should be conducted. We also suggested that population- and meal-specific reference ranges should be employed when interpreting DAS.
